# Association of intraluminal thrombus in thoracoabdominal aortic aneurysms with a blood stasis model

**DOI:** 10.3389/fcvm.2026.1815923

**Published:** 2026-07-02

**Authors:** Guangqiang Zhao, Nan Hu, Peng Wu, Zijian Dong, Kang Han, Xiaoqiang Li, Ran Tao, Xudong Jiang

**Affiliations:** 1 Department of Vascular Surgery, Nanjing Drum Tower Hospital Clinical College of Nanjing University of Chinese Medicine, Nanjing, China; 2 Department of Interventional Therapy, Jining Hospital of Xiyuan Hospital of China Academy of Chinese Medical Sciences, Jining, China; 3 Precision and Intelligence Medical Imaging Lab, Beijing Friendship Hospital, Capital Medical University, Beijing, China; 4 General Surgery Department, The Second Affiliated Hospital of Anhui Medical University, Hefei, China

**Keywords:** blood stasis, Computational Fluid Dynamics (CFD), ILT, OBVF, thoracoabdominal aortic aneurysm

## Abstract

**Objective:**

The aim of this study was to perform patient-specific hemodynamic simulations of thoracoabdominal aortic aneurysm (TAAA) models and evaluate the potential for intraluminal thrombus (ILT) formation.

**Methods:**

Computed Tomography Angiography data from 12 TAAA patients were reconstructed into patient-specific models, which were grouped according to the presence or absence of ILT accumulation. Old blood volume fraction (OBVF) and convergence time were proposed to reveal the association between thrombotic potential and blood stasis in TAAA.

**Results:**

The aneurysm exhibited high blood stasis compared with other parts. In the ILT group, normalized OBVF and convergence time (a passive-tracer washout time, not a physiological thrombosis timescale) were 76.66% ± 12.38% and 42.58 ± 13.29 s, respectively, whereas in the non-ILT group, they were 21.42% ± 3.39% and 10.93 ± 5.96 s. The OBVF and convergence time were significantly greater in the ILT group than in the non-ILT group (*P*-values were <0.0001, <0.01), indicating pronounced blood stasis.

**Conclusion:**

The blood stasis model provides a new and intuitive approach for clinicians to diagnose and treat ILT in TAAA, offering clearer insights than parameters related to WSS. This model can provide useful guidance for operative planning and postoperative evaluation.

## 1 Introduction

Thoracoabdominal aortic aneurysm (TAAA) is a prevalent cardiovascular disorder attributed to multifactorial etiologies, including tobacco exposure, genetic predisposition, and atherosclerotic pathogenesis (1). This clinically silent condition demonstrates asymptomatic progression in most patients, yet carries catastrophic consequences upon rupture, with associated mortality rates surging to approximately 50% (2, 3). Current clinical guidelines recommend prophylactic intervention when aneurysmal dilation exceeds 5 cm in maximal diameter to mitigate rupture risk and optimize therapeutic outcomes (2, 3). Emerging evidence delineates a complex pathophysiological cascade driving aneurysmal degeneration, characterized by phenotypic modulation of vascular smooth muscle cells (VSMCs), leukocytic infiltration, extracellular matrix degradation, and progressive intraluminal thrombus (ILT) formation (4, 5). Notably, ILT has garnered increasing scientific interest due to its dual role in influencing hemodynamic wall stress distribution (6) and perpetuating localized inflammatory responses through cytokine-mediated pathways.

The progression of TAAA is closely linked to complex blood flow changes, primarily influenced by aneurysmal volumetric parameters and morphological characteristics (7). Comparative analyses with normal aortic architecture reveal that aneurysmal lumens exhibit significantly amplified retrograde flow components, characterized by substantially diminished flow velocities and pathologically oscillatory wall shear stress (WSS) patterns (8). ILT significantly impacts aneurysm blood flow patterns by reducing internal circulation and altering nearby vessel structure, which further modifies flow characteristics (9–13). Biomechanical analyses of TAAA rupture mechanisms consistently identify predilection sites exhibiting triad characteristics: critically reduced WSS magnitudes, elevated oscillatory shear index (OSI) values, and substantial ILT accumulation (14, 15). Areas of critical low average wall stress (TAWSS) show accelerated growth (>9 mm/year) and ILT development before rupture (16). These findings emphasize the importance of tracking blood flow patterns and ILT formation to better understand aneurysm progression and improve rupture prediction.

Several studies have confirmed that low TAWSS in aneurysms (17) is associated with the accumulation of ILT (14, 18) and vascular hypoxia (19, 20), leading to localized vascular damage and even rupture. In contrast, other studies have shown that vessels with high WSS levels (>30 dynes/cm^2^) in the AAA are more inclined to rupture (21).

It is generally believed that low TAWSS combined with high OSI promotes ILT formation, which may lead to enlargement or rupture of the AAA; however, it has also been reported that in small-diameter AAA, low OSI levels promote ILT formation in the aneurysm sac, and that when the diameter of the AAA increases, the OSI value does not play a role in ILT deposition (22). Thus, the assessment of ILT using traditional hemodynamic parameters may yield conflicting results. Endothelial Cell Activation Potential (ECAP) (23), defined as the ratio of OSI to TAWSS, characterizes the susceptibility of the arterial wall to thrombosis. Relative Residence Time (RRT) has recently been developed as a new hemodynamic component to characterize the relatively slow blood flow in the vicinity of the aneurysm wall (24, 25). Both novel parameters demonstrate significant predictive potential for thrombogenesis.

Blood stasis is a critical determinant of intra-aneurysmal thrombosis, where reduced flow velocities, impaired metabolite clearance, and hypoxic conditions collectively promote thrombus formation (26, 27). It is well known that Virchow's triad includes three categories of factors that may contribute to thrombosis: hypercoagulability, hemodynamic changes (turbulence or stasis), and endothelial injury/dysfunction. Blood stasis, as an important component, is different from conventional hemodynamic parameters, which only describe the changes in blood flow within the vessel walls. Instead, blood stasis represents a volumetric fluid dynamic state and may have a closer correlation with thrombosis. This investigation implemented a blood stasis model to quantify global flow stagnation, utilizing two-phase flow computational modeling to predict ILT development patterns in TAAAs.

In this study, 12 patients diagnosed with thoracoabdominal aortic aneurysms at Nanjing Drum Tower Hospital were enrolled. Patients were divided into Group A (NILT group, which means without ILT) and Group B (ILT group). Patient-specific models were constructed by collecting CTAs for both groups. Numerical hemodynamic simulations were carried out to simulate blood flushing and stagnation within the aneurysm. The blood stasis model was applied with conventional WSS-related hemodynamic parameters to compare the hemodynamic differences between the two groups, with the aim of exploring the link between ILT formation and blood stasis within thoracoabdominal aortic aneurysms.

## 2 Methods

### 2.1 Patient-specific models

**Figure 1:**
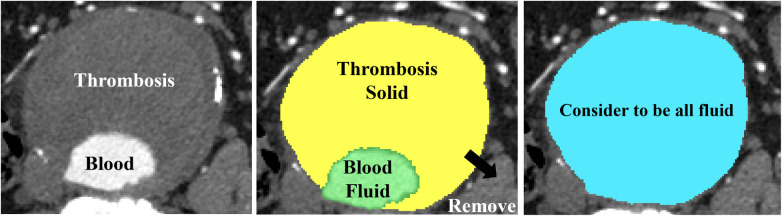
Regions with high Hounsfield unit (HU) values in the CTA slice correspond to blood, while those with low HU values indicate thrombosis. In Mimics software, these two components were individually segmented using distinct masks. The thrombosis “removal” operation entails merging the two mask units and treating the originally solid thrombus as a fluid phase, thus defining the entire aneurysm as a unified fluid domain. By conducting hemodynamic simulations on the region that was originally occupied by solid thrombus, the correlation between blood stasis and ILT formation can be established.

**Figure 2:**
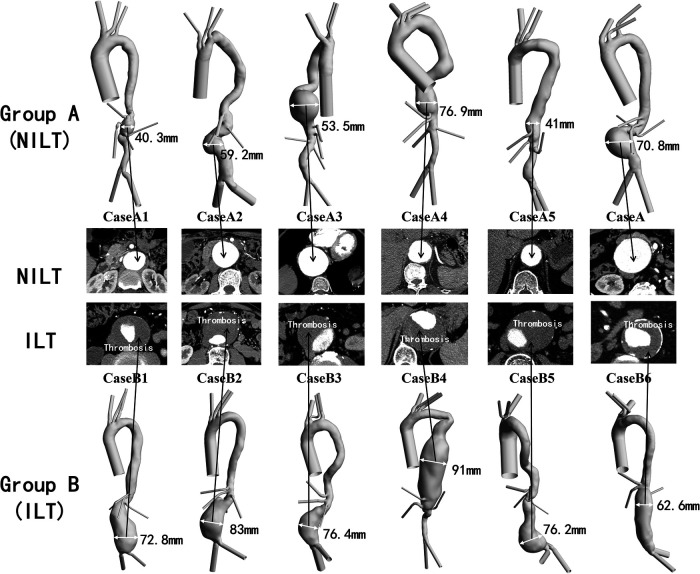
Patient-specific models: CTA shows the largest aneurysm diameter; the thrombotic part was removed during reconstruction and the corresponding part was considered to be the fluid domain.

A total of 12 patient-specific models were reconstructed from CTA images using Mimics 20.0 (Materialise, Plymouth, MI, USA). Surface smoothing was performed in Geomagic 20.0 (3D Systems, Inc., Rock Hill, SC, USA) to enhance the robustness of simulations. Notably, the ILT component in the aneurysm was removed and considered part of the fluid domain. By predicting blood stasis in this region, the risk of thrombosis was assessed, as shown in Figure 1. Patient-specific models and CTA images at the site of maximal aneurysm diameter are shown in Figure 2.

Patient-specific information is summarized in Table 1, including thrombus volume and the ratio of thrombus in the thrombus group. The mean ages of patients in Group A and Group B were 69.17 ± 10.83 years and 67.50 ± 9.65 years, respectively. There was no significant difference in age between the two groups (*P* = 0.784). Differences in gender and aneurysm morphology were also avoided to minimize interference with the results. Notably, aneurysm size remains a key factor affecting rupture and ILT formation. Therefore, the maximum diameters of aneurysms in the thrombus group were larger than those in the non-thrombus group.

**Table 1:** Patient specifications.

Group	Case	Gender	Age (years)	Aneurysm maximum diameter (mm)	Aneurysm volume (cm^3^)	Aneurysm topology	Thrombus volume (cm^3^)	Ratio of thrombus
Group A (NILT)	Case A1	Female	50	40.3	35	Fusiform	—	—
	Case A2	Male	65	59.2	118	Saccular	—	—
	Case A3	Male	77	76.9	163	Saccular	—	—
	Case A4	Male	77	53.5	255	Fusiform	—	—
	Case A5	Male	68	41	42	Fusiform	—	—
	Case A6	Female	78	70.8	169	Saccular	—	—
Group A (ILT)	Case B1	Male	68	72.8	427	Saccular	329	77.05%
	Case B2	Male	62	83	337	Saccular	249	73.89%
	Case B3	Male	81	76.4	306	Saccular	160	52.29%
	Case B4	Male	53	91	694	Fusiform	398	57.35%
	Case B5	Female	67	76.2	236	Saccular	180	76.27%
	Case B6	Male	74	62.6	306	Fusiform	204	58.96%
*P* value	—	—	*P* = 0.784	*P* < 0.05	*P* < 0.01	—	—	—

### 2.2 Mesh and boundary condition

**Figure 3:**
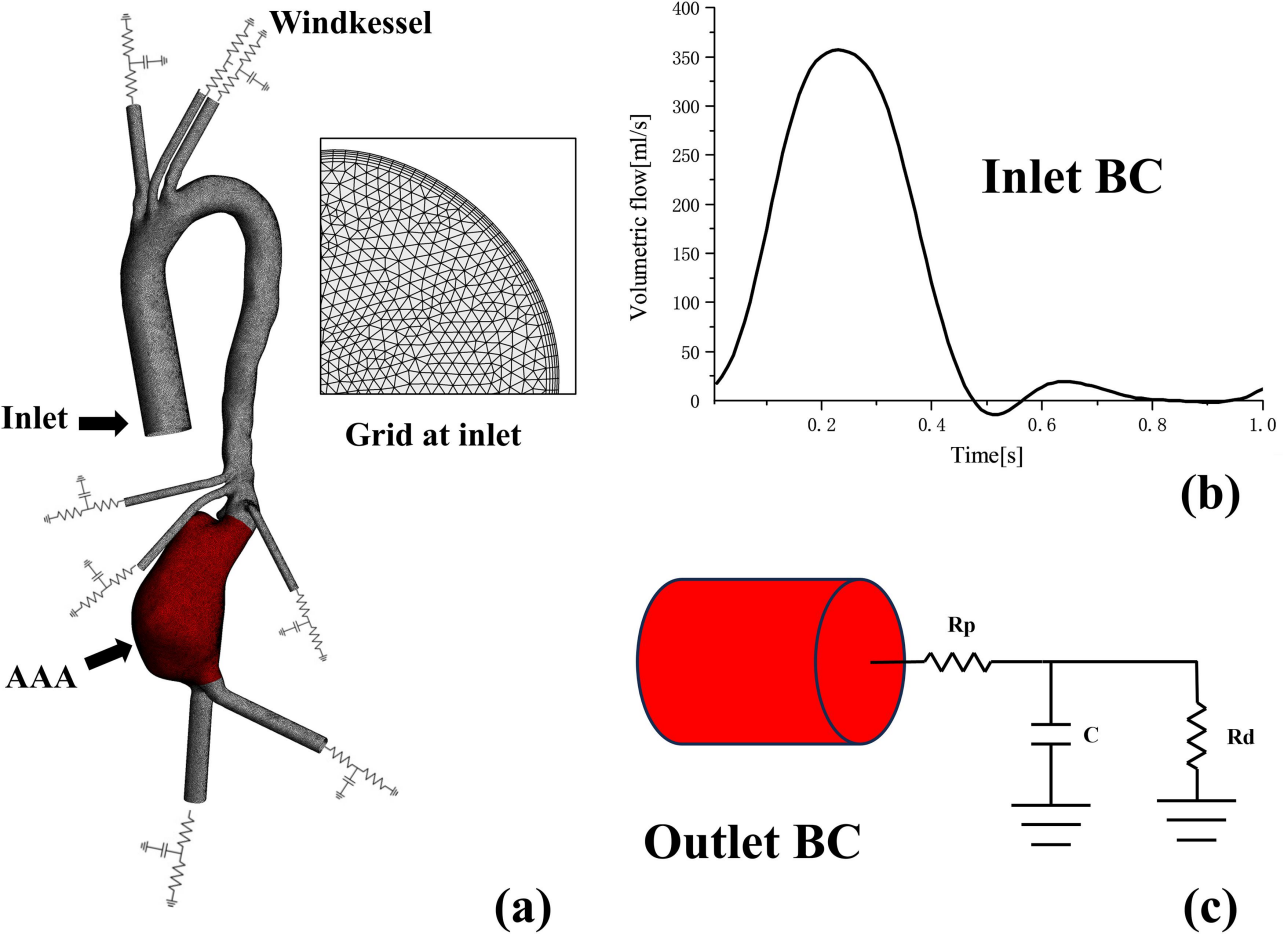
Mesh and boundary condition: **(a)** grids of Case B2, **(b)** the inlet velocity profile, and **(c)** outlet boundary conditions applied with three EWM models.

The aneurysm were divided into separate fluid domains to simulate blood stasis. The inlet was positioned at the aortic root. All inlets and outlets were extended to suppress computational instability, which may arise due to backflow. For mesh sensitivity analysis, three grids of 8.24, 4.57, and 2.18 million elements were generated for Case A1. The grid of 4.57 million elements was confirmed as the appropriate mesh for simulation (see Section 3). Grids of 4.35–6.84 million elements were generated for the remaining 11 models using commercial software Ansys Meshing (Ansys, Inc., Canonsburg, PA, USA), with a similar setup as the 4.57-million grid. Five grid layers were added to all the arterial walls to correctly resolve the boundary layer and guarantee accuracy in simulation, as shown in Figure 3. A time-varying volumetric flow rate, extracted from the literature, was applied at the inlet of each model for a period of 1 s (28). Windkessel Proximal Resistance, Capacitance and Distal Resistance (RCR) boundary conditions were applied at the outlets. Three Element Windkessel Model (EWM) parameters were calculated through patient-specific iteration, with values provided in Supplementary Table S1. All walls were assumed to be rigid with no slip conditions.

### 2.3 Numerical simulation

All single-phase flow simulations in this study were transient and conducted using commercial software Ansys Fluent 22R1 (Ansys, Inc., Canonsburg, PA, USA). Blood was regarded as an incompressible Newtonian fluid, with a density of 1,055 kg/m^3^ and dynamic viscosity of 3.5 × 10^−3^ Pa s. Since the potential presence of turbulence within the aorta (29, 30), the flow was assumed to be turbulent using the k-*ω* Shear-Stress Transport (SST) model. The peak Reynolds numbers ranged from 362 to 1,805, while the mean Reynolds numbers ranged from 55 to 323. The *y*+ values were all close to 1. A second-order implicit backward Euler scheme was chosen for temporal discretization, with a fixed time step of 10 ms, so that each cardiac cycle was resolved using 100 time steps. A maximum of 50 sub-iterations were used for each physical time step, and the maximum RMS residual was set to 10^−5^ as a convergence criterion. Unsteady simulations were carried out for approximately 10 cardiac cycles to obtain statistically converged flow fields, followed by another 10 cardiac cycles to achieve hemodynamic parameters such as TAWSS and RRT.

A two-fluid model was employed to simulate the process of blood stasis, continuing from the converged single-fluid flow field, in accordance with prior studies (26, 27). The computational setup was identical to the single-fluid runs, except that the VOF method was employed to solve the two-fluid flow field. New blood would gradually replace old blood, blood stasis would be tracked and monitored over time. Considering computational resources and time costs, convergence criteria were set that the old blood volume fractions (OBVFs) dropped within 5% in the past 10 cardiac cycles for all cases, and the corresponding time is the convergence time. Differences in blood stasis were evaluated, by comparing convergent OBVF values and convergence times of the two groups, and then correlated with ILT deposition. All computations were performed on a 192-core cluster equipped with 16 Intel Xeon E5-2680 v3 CPUs. Single-fluid simulations normally converged within 4 h, while two-phase flow simulations took less than 2 days.

## 3 Results

### 3.1 Grid sensitivity analysis

Grid sensitivity analysis was conducted for Case A1. The OBVF within the aneurysm at the fifth cardiac cycle was compared to the results predicted with the fine mesh. OBVFs are presented in Table 2. The differences between the results of the “Middle” and “Fine” mesh were almost negligible when compared with the “Coarse” mesh. Therefore, the middle grid was employed for the analyses, and similar setups were employed when generating grids for the remaining cases.

**Table 2:** Results of grid sensitivity analysis.

Mesh	Cells (×10^6^)	OBVF (%)	Error of OBVF (%)
Coarse	2.18	20.3	5.2
Middle	4.57	19.6	1.6
Fine	8.24	19.3	/

Error of OBVF (%), defined as |OBVF-OBVF_0_|/OBVF_0_, where OBVF_0_ is the OBVF of the aneurysm at the fifth cardiac cycle predicted with the fine mesh.

### 3.2 Flow patterns

**Figure 4:**
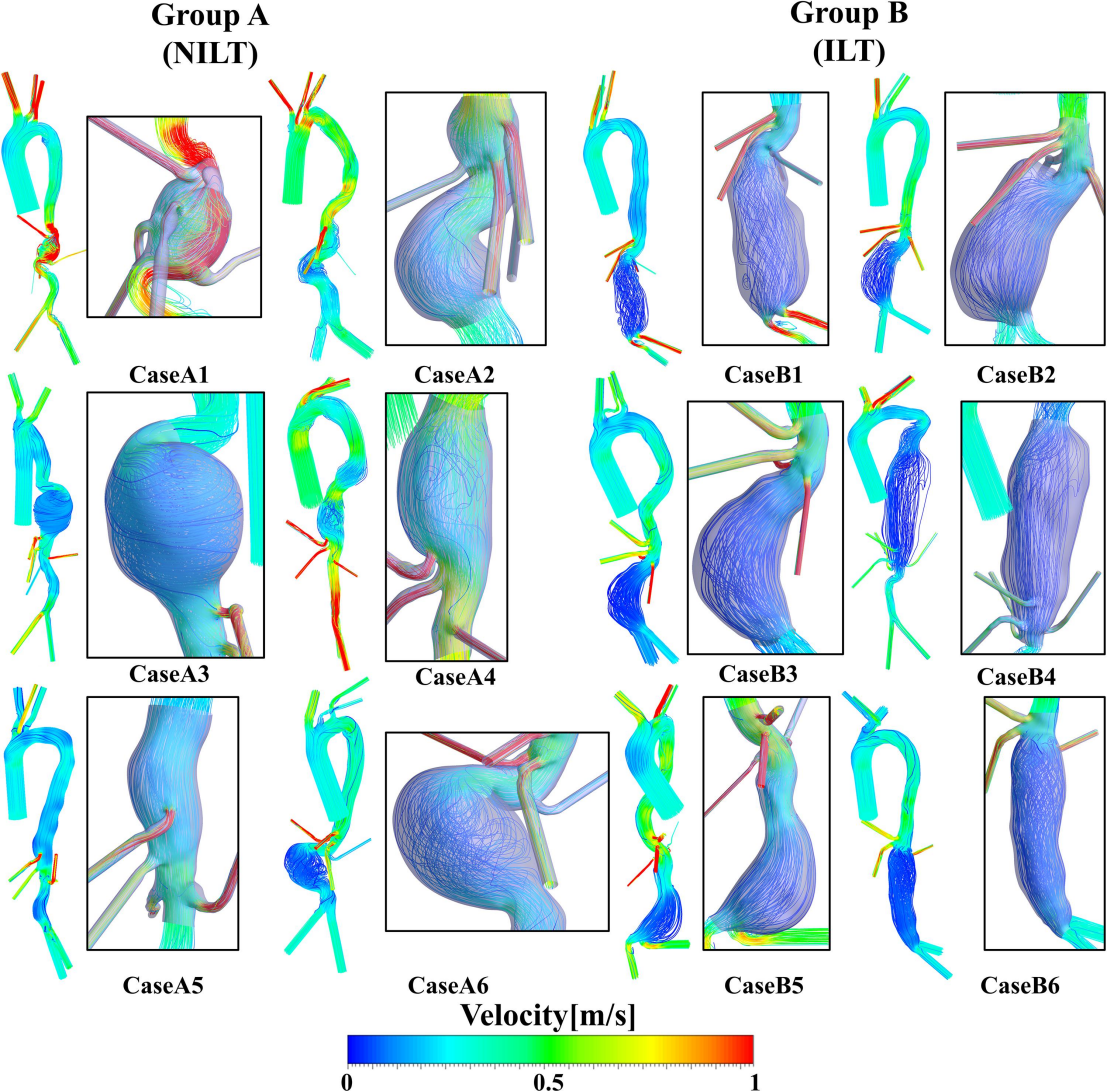
Flow patterns: compared with Group B, the flow rate in aneurysms of Group A was higher (especially in Case A1, where the red flow line appeared in the aneurysm), and the flow field was more chaotic (for example, Case A3 and Case A6, where the main flow in the aneurysm was a vortex).

Blood flow within the aneurysm was slow and dominated by whirlpools, particularly near the entrance, where the flow rate decreased due to dilation of aorta, resulting in reflux and secondary flow. The flow streamlines at the systolic peak of the two groups are shown in Figure 4. By comparing the flow rate of aneurysms in Group A and Group B, it was noted that the velocity in the non-thrombotic group was significantly higher than that in the thrombotic group. Notably, the streamlines of aneurysm in Group B were dark blue, indicating low-velocity conditions, generally less than 0.1 m/s. By observing the flow patterns of these two groups, it was noted that the streamlines in Group A appeared more chaotic than those in Group B, especially in Case A3 and Case A6. In Group A, the main flow in the aneurysm presented a spiral state, and the entire flow field was full of disordered and chaotic multiple eddies. In contrast, the flow pattern in the thrombus group was mainly non-vortex, with only small eddies at the inlet and outlet of the aneurysm, with the flow field being relatively stable.

### 3.3 TAWSS and OSI

**Figure 5:**
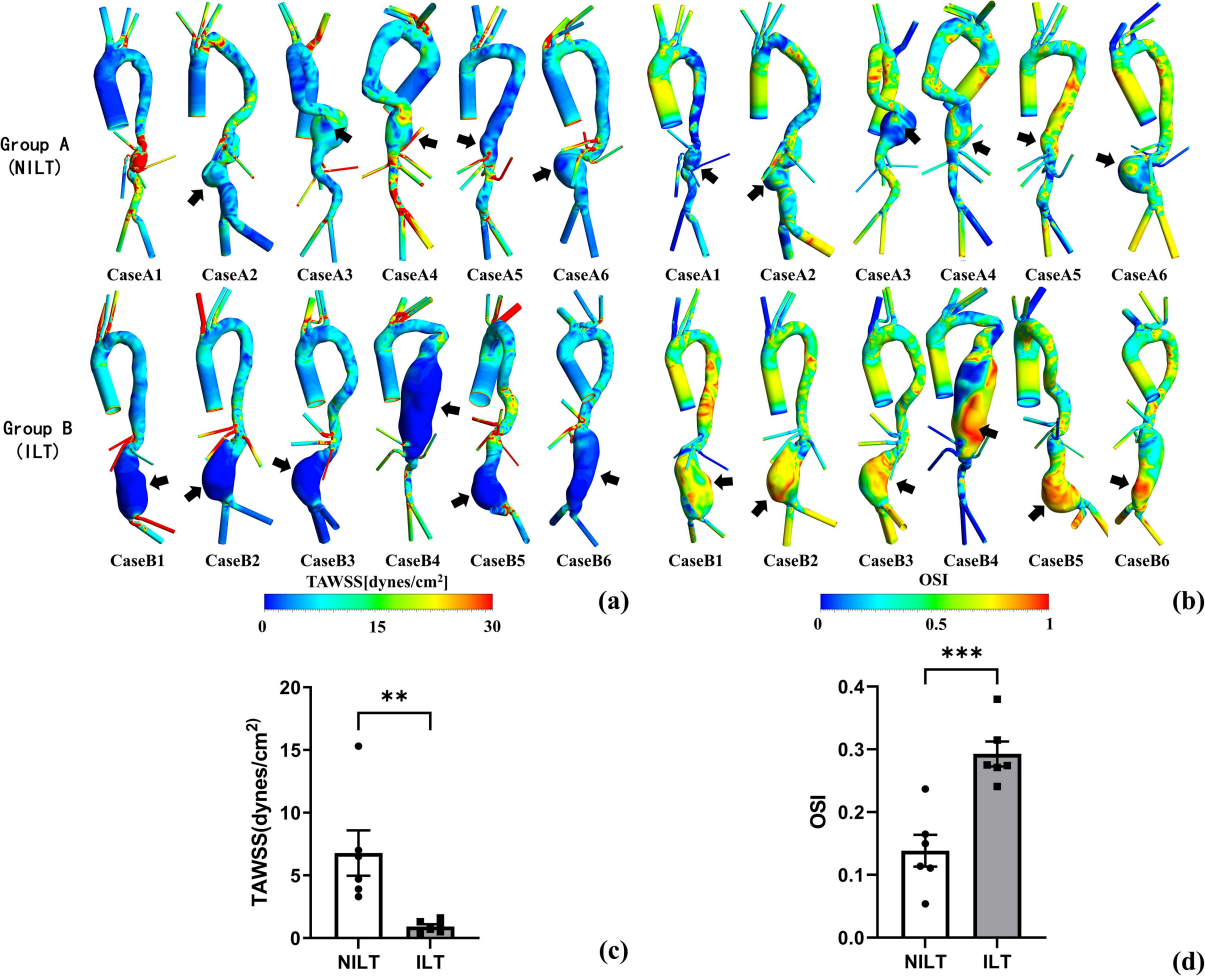
TAWSS and OSI: **(a)** TAWSS contours. Compared with Group A, there was a significantly low TAWSS region in the aneurysm of Group B, as shown by the black arrow. **(b)** OSI contours show that the OSI of aneurysms in the thrombus group was generally higher than that in the non-thrombus group. **(c)** The wall-averaged TAWSS in aneurysm; NILT represents the non-thrombotic group and ILT represents the thrombotic group. The value of non-thrombotic group is significantly larger than the thrombotic group (*P* < 0.01); **(d)** the wall-averaged OSI was significantly higher in the thrombus group than in the non-thrombus group (*P* < 0.001).

TAWSS and OSI are commonly used hemodynamic parameters to assess the risk of aneurysm rupture and thrombosis. It is generally accepted that low TAWSS and high OSI predispose one to thrombosis (31, 32). The distribution of TAWSS and OSI in the two groups is shown in Figures 5a,b, respectively. In the non-thrombotic group, the distribution of TAWSS in aneurysmal regions was not significantly different from that in other regions, and only low-TAWSS areas appeared locally in the aneurysm. Similarly, the overall OSI values across aneurysms in six patients were not significantly increased; in particular, Case A1, Case A3, and Case A6 exhibited low OSI states. Different from Group A, patients in Group B showed a significant decrease of TAWSS across aneurysmal regions, with nearly the entire aneurysm wall of six cases in a state of low TAWSS. On the contrary, large areas of high OSI were observed on the walls of Case B4, Case B5, and Case B6, and the OSI values of the aneurysmal region in the thrombus group were generally higher than those in the non-thrombus group.

Wall-averaged TAWSS and OSI values at the aneurysm were compared between groups, as shown in Figures 5c,d. In this study, an unpaired *t*-test was employed to compare differences between the two datasets. The average TAWSS value of patients in Group A was 6.78 ± 4.41 dynes/cm^2^ and that in group B was 0.92 ± 0.47 dynes/cm^2^. The average TAWSS value in the thrombus group was significantly lower than that in the non-thrombus group (*P* < 0.01). In contrast, the average OSI value in Group A was 0.139 ± 0.062 and that in Group B was 0.293 ± 0.049. The average OSI value in the thrombus group was significantly higher than that in the non-thrombus group (*P* < 0.001).

### 3.4 ECAP and RRT

**Figure 6:**
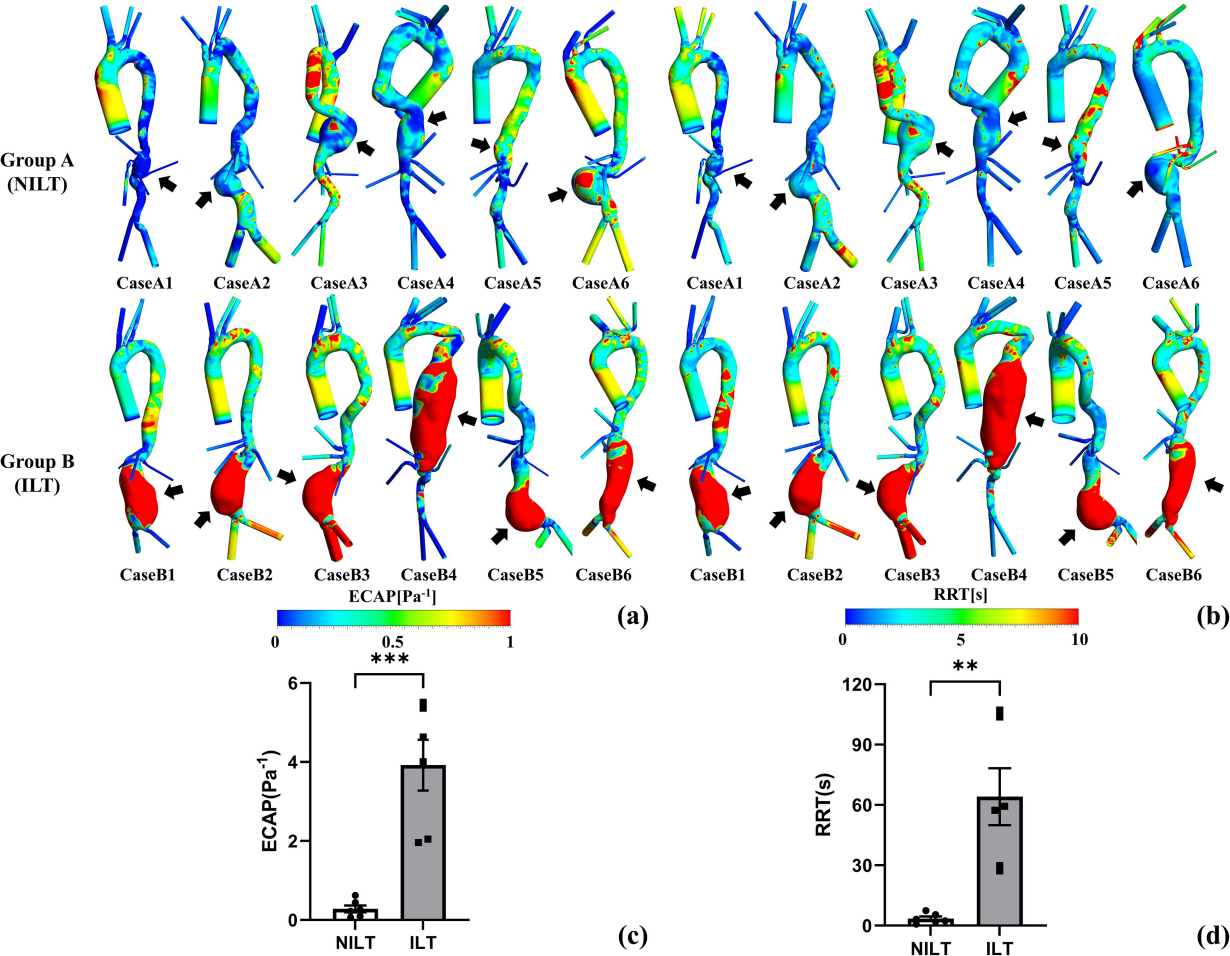
ECAP and RRT: **(a)** ECAP contours: compared with Group A, there was a significantly high ECAP region in the aneurysm of Group B, almost completely covered by red, as shown by the black arrow **(b)** RRT contours: similar to ECAP distribution, the RRT of aneurysms in the thrombus group was generally higher than that in the non-thrombus group; **(c)** for the wall-averaged ECAP in aneurysms, the non-thrombotic group was significantly lower than the thrombotic group (*P* < 0.001); **(d)** the wall-averaged RRT in aneurysm area was significantly higher in the thrombus group than in the non-thrombus group (*P* < 0.01).

ECAP and RRT are hemodynamic parameters related to WSS, and it is generally believed that high ECAP and RRT values suggest higher risk of thrombosis (23–25). As shown in Figures 6a,b, the ECAP and RRT values did not increase significantly in the non-thrombotic group, with only ECAP value of Case A6 being higher in the middle of the aneurysm and ECAP and RRT values of Case A1 and Case A4 being reduced in the aneurysms. In contrast to the non-thrombotic group, the RRT and ECAP values in Group B increased significantly, with entire aneurysms being covered by high RRT and ECAP values.

Wall-averaged ECAP and RRT values in the aneurysmal regions in the two groups are shown in Figures 6c,d. The average ECAP value in the non-thrombotic group (NILT) was 0.28 ± 0.21 Pa^−1^ and that of the thrombus group (ILT) was 3.92 ± 1.58 Pa^−1^. The average ECAP on the aneurysm wall of Group A was significantly lower than that of Group B (*P* < 0.001). Similar to ECAP, the average RRT value in aneurysmal regions of Group A was 3.49 ± 2.48 s and that in Group B was 64.11 ± 34.72 s. The average RRT value of the wall in Group A was also significantly lower than that in Group B (*P* < 0.01). As mentioned previously, the simulation results suggested that the discrimination of ILT by ECAP and RRT values was consistent with the clinical manifestations.

### 3.5 Blood stasis

**Figure 7:**
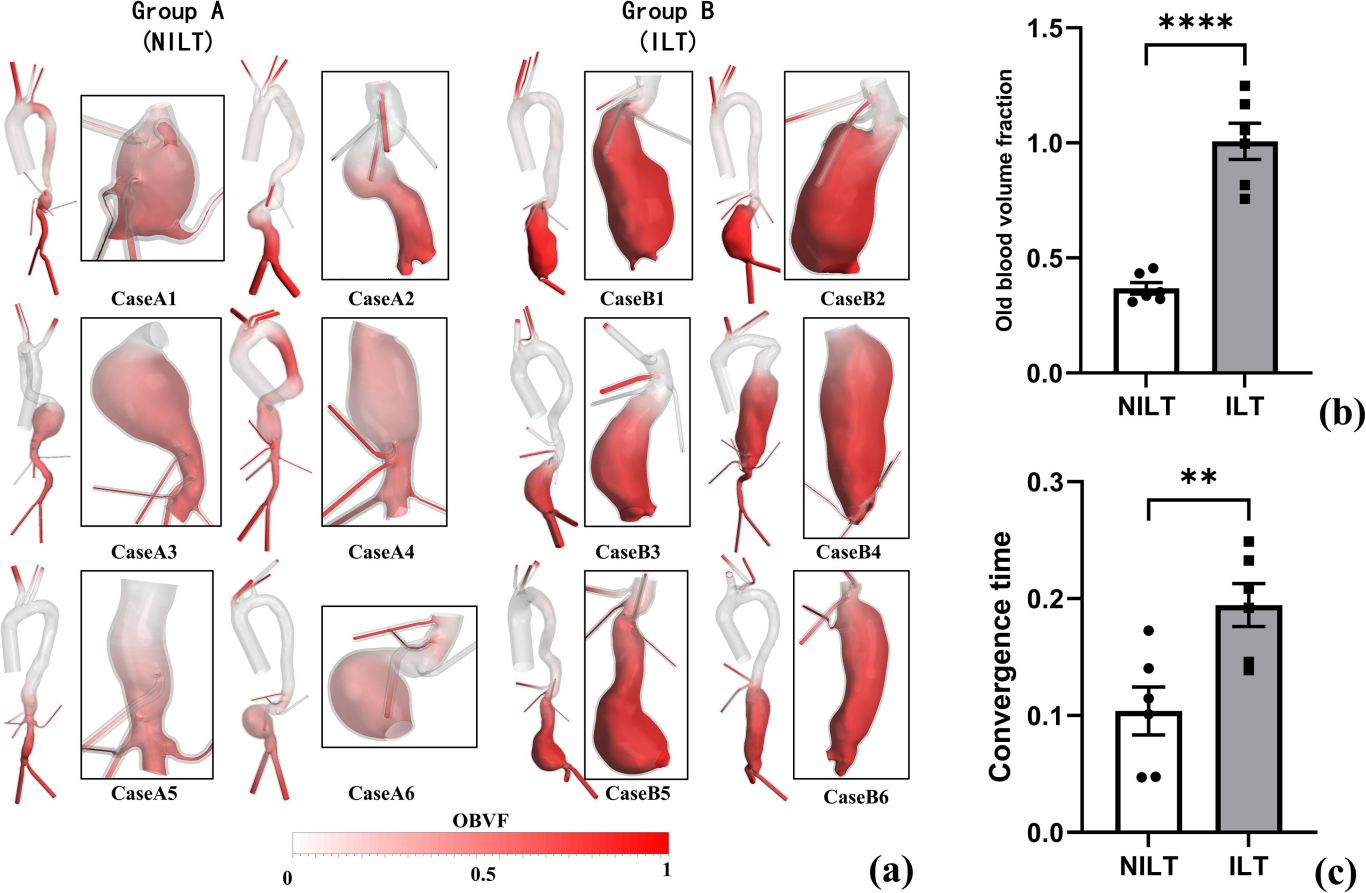
Blood stasis **(a)** convergence OBVF contours show that more old blood remained in the aneurysm of Group B compared with Group A, especially Case B1, Case B2, and Case B5, which were almost dark red, indicating that blood stasis in the thrombus group was higher than that in the non-thrombus group; **(b)** for the convergence OBVF, the non-thrombotic group was significantly smaller than the thrombotic group (*P* < 0.0001); **(c)** for the convergence time, the thrombus group was significantly longer than the non-thrombus group (*P* < 0.01).

Using the two-fluid model, the distribution of old blood was predicted and to characterize blood stasis, as shown in Figure 7a. In both groups, red regions remained in the aneurysms; the thrombus group exhibited a significantly deeper color than the non-thrombus group, particularly in Case B1, Case B2, and Case B5, which meant that blood stasis was more obvious than that in the non-thrombus group.

The OBVF and convergence time for aneurysms in the two groups are shown in Figures 7b,c. To minimize the influence of aneurysm size and maximum diameter, OBVF and convergence time were normalized by diameter and volume, respectively. As shown in Figure 7b, the convergence OBVF of aneurysms in Group A (NILT) was 21.42% ± 3.39% and that in Group B (ILT) was 76.66% ± 12.38%. The OBVF in the non-thrombotic group was significantly lower than that in the thrombotic group (*P* < 0.0001). As shown in Figure 7c, the convergence time of aneurysms in the non-thrombotic group was 10.93 ± 5.96 s and that in the thrombotic group was 42.58 ± 13.29 s. The convergence time in Group A was significantly lower than that in Group B (*P* < 0.001). This simulation demonstrated that the degree of blood stasis in the thrombotic group was significantly higher than that in the non-thrombotic group, which was better associated with the formation of ILT. It can be indicated that the degree and location of thrombosis can be associated well by using blood stasis model.

## 4 Discussion

Blood stasis plays an important role in the thrombosis of thoracic and abdominal aortic aneurysms. Slow flow leads to platelet aggregation and promotes the hypercoagulability of blood, causing a lack of oxygen in the arterial wall. In this study, blood stasis was predicted using a two-fluid model applied to thoracic and abdominal aortic aneurysms. Through hemodynamic simulation of six patients in each group (thrombus versus non-thrombus), it was found that the blood flow in aneurysms of the thrombus group was slow, while that in the non-thrombus group was fast with complex flow patterns. Under such circumstances, the OBVF and convergence time indicated that blood stasis was more severe in the thrombus group.

In previous studies, conventional hemodynamic parameters such as TAWSS and OSI were often used to evaluate the potential of ILT, and it was generally believed that regions with low TAWSS, high OSI, high ECAP, and high RRT were prone to the formation of ILT (19–25). In this study, simulations confirmed that aneurysm walls in the thrombus group had low TAWSS, high OSI, high ECAP, and high RRT. On the contrary, aneurysmal regions in the non-thrombotic group were not significantly different from other areas, and some cases even exhibited high TAWSS and low OSI. The difference in conventional hemodynamic parameters between the two groups demonstrated that the wall-averaged TAWSS in the thrombus group was significantly lower than that in the non-thrombus group (*P* < 0.01), while the wall-averaged OSI, ECAP, and RRT were significantly higher than those in the non-thrombotic group (*P*_OSI_ < 0.001, *P*_ECAP_ < 0.001, *P*_RRT_ < 0.01), consistent with previous research results.

In most studies, the combination of low TAWSS and high OSI has been associated with aneurysm growth and rupture (33–36). In the present study, regions with low TAWSS (<4 dynes/cm^2^) and high OSI (>0.3) were more extensive in the thrombus group than the non-thrombus group, consistent with previous findings. However, some research has reported contrasting results. For example, Arzani and Shadden (37) found that thrombosis commonly formed in regions where TAWSS ranged between 2 and 3 dynes/cm^2^, while OSI was negatively correlated with thrombosis accumulation. Notably, ILT accumulation was not observed in regions with high OSI (>0.4) and low TAWSS (<1 dynes/cm^2^). Other studies have shown that ILT formation can occur in either high or low OSI regions, indicating that OSI may not be directly related to ILT (22, 38). As there are discrepancies in assessing ILT formation using TAWSS and OSI, new metrics are needed to assess ILT formation. In this research, convergence OBVF and convergence time were considered hemodynamic indicators in the blood stasis model. Unlike WSS-related parameters, these two parameters, describing the spatial and temporal characteristics of blood stasis, offered more advantageous associations with ILT formation in aneurysms. It can be observed that OBVF and convergence time were significantly greater in the thrombus group than in the non-thrombus group (*P*_OBVF_ < 0.0001, *P*_convergence time_ < 0.001). More importantly, among the six patients in Group B, there was a high degree of thrombosis (defined as the ratio of volume of ILT and aneurysm) in Case B1, Case B2, and Case B5 (with the degree value above 70%), while the degree of thrombosis in the other three cases was below 60%. The OBVF of the first three patients was above 82%, with values less than 69% for the remaining three patients. It was observed that the difference between OBVF and the degree of thrombosis in each case was less than 15%, while the relevant parameters of WSS could not accurately distinguish variations in the degree of thrombosis. Notably, given the limited sample size, this finding was purely observational and did not reach statistical significance. Nevertheless, this phenomenon still merits further investigation into the correlation between OBVF and the degree of thrombosis.

Blood stasis increases the possibility of platelet adhesion and deposition, leading to thrombosis. In fact, thrombosis is a very complex process, including a series of cascade reactions involving endothelial injury and platelet activation. Nevertheless, this study provided a highly efficient and rapid alternative for evaluating ILT accumulation in TAAA. The blood stasis model can be used to predict the influence of morphological parameters on thrombosis, offering clinicians valuable insights to predict the possibility of ILT formation in TAAA. While RRT describes the relative time of blood retention on the wall of the blood vessel—with high RRT regions prone to thrombosis—blood stasis occurs not only in the wall but also in the overall blood flow field of aneurysms. Therefore, OBVF, as a parameter to describe the volume of blood stasis, is more intuitive than RRT in associating with thrombosis potential. Although RRT can indicate the location of ILT, it cannot distinguish the degree of thrombus formation across different patients. However, the OBVF values of individual patients within the group exhibited a strong correlation with the thrombosis ratios. In addition, recent studies have proposed various ways to describe blood stasis. Rayz et al. (39) employed washout time within basilar aneurysms as a metric to assess blood stasis, which was subsequently compared against measurements derived from 4D flow, and the convergence time represents a continuation and extension of that concept. In the field of imaging, researchers analyzed the radiomics of CT to describe blood stasis and thus predicted the formation of left atrial appendage thrombosis (40, 41). Mathews et al. (42) described blood stasis in deep veins of lower extremities through platelet count, which was associated with deep venous thrombosis. These studies can provide references for the improvement and optimization of the blood stasis model.

This study has several limitations. First, this research predicted blood stasis rather than directly simulating the process of ILT. Therefore, the convergence time only reflected the time scale of old blood being replaced by new blood. Precise thrombosis simulation is needed to consider the various factors leading to thrombosis and predict the growth of thrombosis on a longer time scale, such as 6 months or 1 year. Therefore, the blood stasis model is currently unable to predict the specific time required for thrombosis formation, and more accurate results are needed, with more patients and longer follow-up observations. Furthermore, an expanded sample size would facilitate validation of the model's sensitivity and its correlation with thrombosis. Second, thrombosis is a very complex process, and this study only considered one element of Virchow's triad, namely, slow and stagnant blood flow, without considering platelet changes and the activation process caused by biochemical factors. The time scale for thrombosis was different from the time scale considered in the present study, but this model was nevertheless sufficient to distinguish the risk of thrombosis in different patients. Third, the non-Newtonian properties of blood are equally non-negligible; shear-thinning effects elevate viscosity in low-shear regions and exacerbate blood stasis—particularly in large-volume aneurysms with low WSS. In future research, non-Newtonian models such as Carreau-Yasuda or Casson should be incorporated into two-phase flow simulations to improve the precision of the blood stasis model. Finally, all simulations were based on CTA images from one period. By collecting images at more time points for clinical follow-up, the process of thrombosis can also be better tracked, offering a more precise reference for thrombosis. Moreover, as a retrospective study, patient-specific waveforms were not available. Future work should include patient-specific measured flow rates and sensitivity analyses for boundary conditions.

In summary, through the hemodynamic simulations of thoracoabdominal aortic aneurysms in the thrombotic and non-thrombotic groups, this study demonstrated that the blood stasis model can provide better associations with ILT formation in TAAA compared with WSS-related parameters, and can directly reflect both the temporal and spatial distribution of ILT. By directly associating thrombosis potential with blood stasis, this model provides a new approach for clinicians to diagnose and treat thrombus in TAAA. It can be used for preoperative evaluation and prognosis assessment of more diseases with complex hemodynamic changes, providing valuable guidance for operative planning and postoperative evaluation.

## Data Availability

The original contributions presented in the study are included in the article/Supplementary Material, further inquiries can be directed to the corresponding authors.
