# Supplementary material for: Association of intraluminal thrombus in thoracoabdominal aortic aneurysms with a blood stasis model
Source: Front Cardiovasc Med. 2026 Jul 2;13:1815923. doi: 10.3389/fcvm.2026.1815923 (PMC13373046; doi:10.3389/fcvm.2026.1815923)
Supplement: Supplementary file 1 [file Datasheet1.docx]

Supplementary Table 1 The value of 3 EWM parameters for CaseA1

|  | BCT | LCA | LSA | CA | SMA | LRA | RRA | RIA | LIA |
| --- | --- | --- | --- | --- | --- | --- | --- | --- | --- |
| Rp  （10^7^Pa·s·m^-3^） | 4.7534 | 6.7431 | 0.7155 | 3.8277 | 5.7789 | 4.8465 | 64.8176 | 4.4236 | 9.3153 |
| Rd  （10^8^Pa·s·m^-3^） | 3.9465 | 20.1443 | 7.7249 | 21.2291 | 21.6243 | 66.6241 | 50.1307 | 8.8469 | 8.7507 |
| C  （10^-10^Pa^-1^·m^3^） | 40.4808 | 8.8562 | 22.9591 | 8.2825 | 8.0623 | 2.6673 | 3.1618 | 19.2696 | 18.4874 |

Supplementary Table 2 The value of 3 EWM parameters for CaseA2

|  | BCT | LCA | LSA | CA | SMA | LRA | RRA | RIA | LIA |
| --- | --- | --- | --- | --- | --- | --- | --- | --- | --- |
| Rp  （10^7^Pa·s·m^-3^） | 0.8938 | 1.8460 | 1.9737 | 3.4926 | 16.2779 | 17.9617 | 202.6987 | 16.3617 | 14.1858 |
| Rd  （10^8^Pa·s·m^-3^） | 5.5272 | 26.1713 | 9.2855 | 26.0632 | 26.7479 | 95.4811 | 118.7622 | 6.8456 | 10.2520 |
| C  （10^-10^Pa^-1^·m^3^） | 31.8699 | 6.7916 | 18.8760 | 6.7771 | 6.3082 | 1.8401 | 1.2875 | 2.1104 | 15.3377 |

Supplementary Table 3 The value of 3 EWM parameters for CaseA3

|  | BCT | LCA | LSA | CA | SMA | LRA | RRA | RIA | LIA |
| --- | --- | --- | --- | --- | --- | --- | --- | --- | --- |
| Rp  （10^7^Pa·s·m^-3^） | 0.7581 | 1.3393 | 1.4337 | 0.3990 | 9.8792 | 3.4208 | 101.3859 | 7.5464 | 8.3723 |
| Rd  （10^8^Pa·s·m^-3^） | 5.6540 | 13.3827 | 13.9246 | 16.4199 | 9.9718 | 27.9691 | 41.7749 | 6.6987 | 9.0767 |
| C  （10^-10^Pa^-1^·m^3^） | 31.2403 | 13.2429 | 12.7240 | 10.8750 | 16.3325 | 6.3248 | 3.4480 | 24.0161 | 18.0554 |

Supplementary Table 4 The value of 3 EWM parameters for CaseA4

|  | BCT | LCA | LSA | CA | SMA | LRA | RRA | RIA | LIA |
| --- | --- | --- | --- | --- | --- | --- | --- | --- | --- |
| Rp  （10^7^Pa·s·m^-3^） | 0.1796 | 0.9988 | 0.1596 | 2.2198 | 6.7205 | 1.0786 | 17.8805 | 16.9854 | 15.7179 |
| Rd  （10^8^Pa·s·m^-3^） | 5.4861 | 15.0145 | 21.9018 | 11.9639 | 10.0996 | 24.7175 | 18.1570 | 9.4990 | 7.2396 |
| C  （10^-10^Pa^-1^·m^3^） | 32.5214 | 11.8430 | 8.1669 | 14.6892 | 16.6177 | 7.2104 | 8.9746 | 15.9857 | 20.3146 |

Supplementary Table 5 The value of 3 EWM parameters for CaseA5

|  | BCT | LCA | LSA | CA | SMA | LRA | RRA | RIA | LIA |
| --- | --- | --- | --- | --- | --- | --- | --- | --- | --- |
| Rp  （10^7^Pa·s·m^-3^） | 1.2329 | 0.6384 | 0.5872 | 1.1119 | 8.9349 | 3.9169 | 69.0270 | 4.0366 | 8.8152 |
| Rd  （10^8^Pa·s·m^-3^） | 7.0224 | 24.1495 | 17.6501 | 6.9430 | 16.5301 | 31.1317 | 48.0107 | 5.7201 | 8.6115 |
| C  （10^-10^Pa^-1^·m^3^） | 25.0501 | 7.3926 | 10.1074 | 25.3750 | 10.2734 | 5.6783 | 3.2597 | 29.2306 | 18.8560 |

Supplementary Table 6 The value of 3 EWM parameters for CaseA6

|  | BCT | LCA | LSA | CA | SMA | LRA | RRA | RIA | LIA |
| --- | --- | --- | --- | --- | --- | --- | --- | --- | --- |
| Rp  （10^7^Pa·s·m^-3^） | 1.3340 | 1.5654 | 1.2702 | 3.3223 | 0.2301 | 4.7960 | 95.3344 | 9.8021 | 11.4274 |
| Rd  （10^8^Pa·s·m^-3^） | 8.8190 | 22.1948 | 16.0981 | 6.4204 | 20.2125 | 42.1452 | 50.8636 | 6.7677 | 8.8481 |
| C  （10^-10^Pa^-1^·m^3^） | 19.9947 | 8.0085 | 11.0323 | 26.5080 | 8.8458 | 4.1994 | 2.9637 | 23.1030 | 17.9164 |

Supplementary Table 7 The value of 3 EWM parameters for CaseB1

|  | BCT | LCA | LSA | CA | SMA | LRA | RRA | RIA | LIA |
| --- | --- | --- | --- | --- | --- | --- | --- | --- | --- |
| Rp  （10^7^Pa·s·m^-3^） | 0.5214 | 0.7515 | 5.1837 | 3.6216 | 8.0172 | 18.9113 | 83.6158 | 2.0083 | 7.6635 |
| Rd  （10^8^Pa·s·m^-3^） | 5.0959 | 11.8338 | 5.6203 | 22.1038 | 11.0740 | 32.5670 | 38.9795 | 7.4533 | 4.8232 |
| C  （10^-10^Pa^-1^·m^3^） | 34.7706 | 15.0307 | 29.1584 | 7.9676 | 15.0728 | 5.1947 | 3.7811 | 23.3860 | 32.0238 |

Supplementary Table 8 The value of 3 EWM parameters for CaseB2

|  | BCT | LCA | LSA | CA | SMA | LRA | RRA | RIA | LIA |
| --- | --- | --- | --- | --- | --- | --- | --- | --- | --- |
| Rp  （10^7^Pa·s·m^-3^） | 1.4151 | 0.8964 | 0.8659 | 1.7317 | 6.8934 | 2.6268 | 52.0562 | 8.5878 | 8.7115 |
| Rd  （10^8^Pa·s·m^-3^） | 8.3487 | 12.5992 | 7.8394 | 18.4575 | 12.1055 | 33.8396 | 34.0243 | 9.3756 | 3.1721 |
| C  （10^-10^Pa^-1^·m^3^） | 21.0832 | 14.1069 | 22.5862 | 9.6078 | 13.9900 | 5.2489 | 4.5628 | 17.4901 | 44.2719 |

Supplementary Table 9 The value of 3 EWM parameters for CaseB3

|  | BCT | LCA | LSA | CA | SMA | LRA | RRA | RIA | LIA |
| --- | --- | --- | --- | --- | --- | --- | --- | --- | --- |
| Rp  （10^7^Pa·s·m^-3^） | 0.8100 | 0.4163 | 0.3139 | 8.4694 | 8.4242 | 2.8169 | 51.1385 | 13.0127 | 26.3207 |
| Rd  （10^8^Pa·s·m^-3^） | 5.1041 | 14.5429 | 9.8081 | 13.3206 | 13.4998 | 45.7176 | 14.1459 | 5.8331 | 69.8153 |
| C  （10^-10^Pa^-1^·m^3^） | 34.5217 | 12.2733 | 18.1920 | 12.6345 | 12.4807 | 3.8914 | 9.2940 | 25.0898 | 2.4708 |

Supplementary Table 10 The value of 3 EWM parameters for CaseB4

|  | BCT | LCA | LSA | CA | SMA | LRA | RRA | RIA | LIA |
| --- | --- | --- | --- | --- | --- | --- | --- | --- | --- |
| Rp  （10^7^Pa·s·m^-3^） | 0.4136 | 1.0893 | 0.3139 | 3.2771 | 8.1356 | 11.0683 | 59.2156 | 13.5022 | 16.7553 |
| Rd  （10^8^Pa·s·m^-3^） | 5.8101 | 15.1321 | 14.5575 | 17.1764 | 17.6539 | 52.6473 | 20.3917 | 8.4210 | 75.0214 |
| C  （10^-10^Pa^-1^·m^3^） | 30.5906 | 11.7446 | 12.2696 | 10.2262 | 9.6927 | 3.3300 | 6.8027 | 23.0338 | 2.3339 |

Supplementary Table 11 The value of 3 EWM parameters for CaseB5

|  | BCT | LCA | LSA | CA | SMA | LRA | RRA | RIA | LIA |
| --- | --- | --- | --- | --- | --- | --- | --- | --- | --- |
| Rp  （10^7^Pa·s·m^-3^） | 5.0128 | 0.8233 | 0.9988 | 4.2666 | 7.7872 | 19.3981 | 51.2702 | 11.0949 | 130.7989 |
| Rd  （10^8^Pa·s·m^-3^） | 5.0470 | 13.7965 | 7.6518 | 8.6316 | 8.4924 | 25.2832 | 40.9137 | 7.4984 | 166.1373 |
| C  （10^-10^Pa^-1^·m^3^） | 32.2626 | 12.8974 | 23.0919 | 19.7610 | 19.3072 | 6.5753 | 3.8879 | 20.7949 | 0.9988 |

Supplementary Table 12 The value of 3 EWM parameters for CaseB6

|  | BCT | LCA | LSA | CA | SMA | LRA | RRA | RIA | LIA |
| --- | --- | --- | --- | --- | --- | --- | --- | --- | --- |
| Rp  （10^7^Pa·s·m^-3^） | 1.5468 | 1.9870 | 2.5376 | 1.9963 | 11.8969 | 2.0668 | 50.7235 | 16.6356 | 5.7935 |
| Rd  （10^8^Pa·s·m^-3^） | 4.8589 | 14.1444 | 6.4670 | 9.3693 | 8.9462 | 30.5196 | 35.9722 | 11.7357 | 10.5002 |
| C  （10^-10^Pa^-1^·m^3^） | 35.7031 | 12.4799 | 26.6339 | 18.7063 | 17.6599 | 5.8256 | 4.3611 | 13.3590 | 16.1559 |
